# Retraction: Determination of the Efficiency of Magnetic Resonance Imaging in the Evaluation of Compressive Myelopathy

**DOI:** 10.7759/cureus.r145

**Published:** 2024-07-17

**Authors:** Challa Anil Kumar, Satyanarayana Kummari, Bagadi Lava Kumar

**Affiliations:** 1 Department of Radiodiagnosis, Great Eastern Medical School & Hospital, Srikakulam, Andhra Pradesh, IND; 2 Department of Radiodiagnosis, All India Institute of Medical Sciences, Nagpur, Maharashtra, IND

This article has been retracted by the Editors-in-Chief due to plagiarism. Several images (details below) have been taken from the following published article: https://www.jaypeejournals.com/doi/IJAIMS/pdf/10.5005/jp-journals-10050-10091, Agarwal M, Kumar P, Gupta D. Role of Magnetic Resonance Imaging in the Evaluation of Compressive Myelopathy in Rohilkhand Region, India. Int J Adv Integ Med Sci 2017;2(3):130-136.

- Figure 1A = Figure 2 (right) in Agarwal M, et al.
- Figure 1B = Figure 2 (left) in Agarwal M, et al.
- Figure 4 (all images) = Figure 4 (all images) in Agarwal M, et al.
- Figure 9C = Figure 3B in Agarwal M, et al.

